# Mycophenolate mofetil as salvage therapy for refractory acute-onset CIDP associated with rheumatoid arthritis: a case report

**DOI:** 10.3389/fimmu.2026.1869194

**Published:** 2026-09-30

**Authors:** Jinhui Tan, Hai Huang, Linghua Tan, Bo Li

**Affiliations:** 1 Department of Rheumatology and Immunology, People’s Hospital of Longhua, Shenzhen, Shenzhen, Guangdong, China; 2 Department of Health Management, People’s Hospital of Longhua, Shenzhen, Shenzhen, Guangdong, China; 3 Department of Health Management, Jiangmen Wuyi Hospital of Chinese Medicine, Jiangmen, Guangdong, China

**Keywords:** arthritis, rheumatoid, polyradiculoneuropathy, chronic inflammatory demyelinating, mycophenolate mofetil, drug resistance, immunosuppressive agents

## Abstract

Chronic inflammatory demyelinating polyradiculoneuropathy (CIDP) is an immune-mediated neuropathy that rarely co-occurs with rheumatoid arthritis (RA). When CIDP proves refractory to first-line therapies, management becomes particularly challenging, especially in patients with multiple autoimmune conditions. A 43-year-old Chinese male with a 3-year history of seropositive RA in sustained remission presented with acute progressive quadriparesis, bilateral ptosis, and facial diplegia. Cerebrospinal fluid analysis revealed albuminocytologic dissociation, and nerve conduction studies demonstrated evolving demyelinating features, consistent with a diagnosis of acute-onset CIDP (A-CIDP). The patient responded inadequately to sequential treatment with intravenous immunoglobulin, high-dose corticosteroids, and the neonatal Fc receptor (FcRn) antagonist efgartigimod, experiencing repeated relapses during corticosteroid tapering. Mycophenolate mofetil (MMF) was subsequently introduced as a steroid-sparing agent at 0.75 g twice daily, enabling further prednisone tapering from 20 mg to 10 mg daily without relapse; MMF was later up-titrated to 1 g twice daily. Under a combined, multi-target immunosuppressive regimen of MMF, prednisone, methotrexate, iguratimod, and hydroxychloroquine, the patient achieved sustained neurological improvement, with prednisone successfully tapered to 10 mg daily by October 2025 and maintained thereafter. By April 2026, only residual mild ptosis persisted, and no infectious complications occurred. This case suggests that MMF-based multi-target immunosuppression may be a therapeutic option for RA-associated, treatment-refractory A-CIDP, potentially enabling successful glucocorticoid tapering even after an inadequate response to a short course of FcRn-targeted therapy.

## 1 Introduction

Rheumatoid arthritis (RA) is a chronic, systemic autoimmune disease characterized by symmetrical polyarthritis, progressive joint destruction, and a broad spectrum of extra-articular manifestations (1, 2). Although articular involvement dominates the clinical picture, peripheral nervous system complications are well recognized and include entrapment neuropathies, distal symmetric axonal polyneuropathy, and vasculitic multiple mononeuropathy (1, 2). However, the co-occurrence of RA with chronic inflammatory demyelinating polyradiculoneuropathy (CIDP)—a treatable but potentially disabling immune-mediated neuropathy of the peripheral nerves and nerve roots—has rarely been reported (3, 4).

CIDP is the most common chronic autoimmune neuropathy, with an estimated prevalence of approximately 0.7 to 10.3 per 100,000 population worldwide (5, 6). It typically presents with progressive or relapsing symmetric proximal and distal weakness, sensory loss, and diminished or absent deep tendon reflexes, evolving over at least 8 weeks (7). The 2021 European Academy of Neurology/Peripheral Nerve Society (EAN/PNS) guidelines distinguish typical CIDP from several well-characterized variants, including distal, multifocal, focal, motor, and sensory forms (7). Both humoral and cell-mediated immune mechanisms are implicated in its pathogenesis (6, 8), and first-line therapies—intravenous immunoglobulin (IVIG), corticosteroids, and plasma exchange—achieve symptomatic improvement in approximately 60–80% of patients (9–11). Approximately 15% of patients, however, remain refractory to all conventional treatment modalities, and even patients with initial responses frequently experience relapses or become dependent on high-dose corticosteroids (10, 11).

The coexistence of CIDP with systemic rheumatic diseases, including RA, is exceedingly rare and has been attributed to shared genetic predispositions and overlapping dysregulation of both T-cell and B-cell compartments (3, 4, 12, 13). A 2017 case report described Lewis–Sumner syndrome, a multifocal variant of CIDP, developing almost concurrently with RA, raising the question of shared immunopathological mechanisms (3). The co-occurrence of immune-mediated neuropathies with systemic connective tissue diseases (CTDs), such as systemic lupus erythematosus and Sjögren’s syndrome (SS), is increasingly recognized. For instance, Guillain-Barré syndrome (GBS), an acute monophasic neuropathy, has been reported in association with various CTDs, including systemic lupus erythematosus (SLE) and primary SS (14, 15), where it often presents with atypical features and a potentially poorer prognosis (16, 17). A systematic review of SLE-related GBS found that over half of the patients presented with GBS before their SLE diagnosis, and the condition often required immunosuppressive therapy beyond standard GBS treatment (15). Although GBS and CIDP are distinct entities with different diagnostic criteria and typical time courses (17), they share overlapping immunopathogenetic mechanisms and therapeutic approaches, including the use of intravenous immunoglobulin and, in refractory cases, immunosuppressive agents (16, 17).

When CIDP presents with an acute onset—as occurs in up to 13% of cases (acute-onset CIDP, A-CIDP)—it may initially be misdiagnosed as GBS, with deterioration beyond 8 weeks or repeated relapses ultimately prompting diagnostic revision (7, 18).

Here, we report a challenging case of a patient with seropositive, long-standing RA in sustained clinical remission on conventional disease-modifying antirheumatic drugs, who developed A-CIDP. The disease was refractory to sequential treatment with IVIG, high-dose corticosteroids, and the neonatal Fc receptor antagonist efgartigimod, but was ultimately controlled with MMF as part of a multi-target immunosuppressive regimen. This case highlights the diagnostic and therapeutic challenges of managing CIDP in patients with coexisting autoimmune disease, underscores the potential of conventional immunosuppressive intensification as a salvage strategy, and contributes to the limited literature on this rare clinical association.

## 2 Case presentation

A 43-year-old Chinese male with a 3-year history of seropositive rheumatoid arthritis (RA) presented to the neurology outpatient clinic in August 2024 with acute progressive quadriparesis and bilateral ptosis and was directly admitted to the neurology inpatient department. His RA had been diagnosed in February 2021 at the Longhua District People’s Hospital of Shenzhen, based on symmetric polyarthritis involving the proximal interphalangeal, metacarpophalangeal, and wrist joints, accompanied by prolonged morning stiffness exceeding one hour. Laboratory investigations at diagnosis revealed elevated inflammatory markers (erythrocyte sedimentation rate [ESR] 26 mm/h, C-reactive protein [CRP] 27.1 mg/L), strongly positive anti-cyclic citrullinated peptide antibody (>200 U/mL), rheumatoid factor 179.3 IU/mL, and positive anti-keratin antibody. Joint ultrasonography confirmed active synovitis. He was initially treated with prednisone 10 mg daily, methotrexate 10 mg weekly, hydroxychloroquine 200 mg daily, and sulfasalazine 1 g twice daily. Due to persistent articular symptoms, the treatment was modified in March 2021: hydroxychloroquine and sulfasalazine were discontinued, and iguratimod 25 mg twice daily plus celecoxib 200 mg twice daily were added. By July 2021, complete clinical remission of RA had been achieved, and prednisone was successfully discontinued by August 2021. For the subsequent three years, he remained in sustained RA remission on a maintenance regimen of methotrexate 10 mg weekly and iguratimod 25 mg twice daily.

On July 24, 2024, the patient underwent elective facial cosmetic injections of hyaluronic acid and botulinum toxin (specific product details unavailable). Five days post-procedure, on July 29, he developed insidious-onset fatigue and limb weakness, initially manifesting as difficulty with shoulder abduction and ambulation. The symptoms progressed steadily: by August 2, 2024, he exhibited bilateral ptosis, which was markedly asymmetric with the left side more severely affected, bilateral lagophthalmos, left-sided peripheral facial droop, masticatory muscle weakness, blurred vision, and intermittent dysphagia. There was no diurnal fluctuation, no sensory complaints, and no headache or gait ataxia.

On admission, neurological examination revealed a conscious and oriented patient with mild dysarthria. Cranial nerve examination demonstrated bilateral ptosis (left > right), bilateral lagophthalmos, loss of forehead wrinkling, shallowing of the left nasolabial fold, uvular deviation to the right, and a diminished left gag reflex. Tongue protrusion was midline, and extraocular movements were full without nystagmus. Motor examination revealed bilateral upper limb strength of 5-/5 and bilateral lower limb strength of 4/5. Muscle tone was normal in all four limbs. Deep tendon reflexes were globally diminished. Abdominal reflexes were preserved. Plantar responses were flexor bilaterally. Sensory examination suggested questionable hypoesthesia below both knees. Coordination testing was normal. There was no neck stiffness, and meningeal signs were absent.

Brain magnetic resonance imaging (MRI) with magnetic resonance angiography and venography was unremarkable. A lumbar puncture was performed on August 12, 2024, yielding clear and colorless cerebrospinal fluid (CSF) under an opening pressure of 125 mmH_2_O. CSF analysis revealed marked albuminocytologic dissociation: protein 620 mg/L (elevated), glucose 4.00 mmol/L (normal), and white cell count within normal limits. Routine CSF studies, bacterial and fungal cultures, acid-fast bacillus smear, and cryptococcal antigen were all negative. Serum testing for anti-acetylcholine receptor and anti-muscle-specific kinase antibodies was negative, as was serum testing for botulinum toxin neutralizing antibodies (using the mouse neutralization bioassay). Furthermore, the patient’s clinical presentation, featuring ascending and asymmetric weakness, was inconsistent with the typical descending and symmetric flaccid paralysis seen in botulism, effectively ruling out systemic botulinum toxin effect as the primary etiology. The neostigmine test was equivocal. A comprehensive serum anti-ganglioside antibody panel (24 items) was positive for GM4-IgG. The initial nerve conduction study, performed on August 9, 2024, was unremarkable except for equivocal low-frequency decrement on right facial nerve repetitive stimulation; all limb nerve conduction velocities, distal latencies, and compound muscle action potential (CMAP) amplitudes were within normal limits.

An initial diagnosis of acute GBS was made; however, given the diagnostic uncertainty between GBS and A-CIDP, the patient received a 5-day course of intravenous immunoglobulin (25 g daily) combined with pulsed intravenous methylprednisolone (500 mg/day for 4 days, tapered sequentially to 250 mg/day for 2 days, then 125 mg/day for 1 day) to cover both potential disease entities. Despite this treatment, significant bilateral ptosis, facial diplegia, and quadriparesis persisted, with only marginal subjective improvement. He was discharged on August 23, 2024, on oral prednisone 60 mg daily with a planned slow taper, alongside RA maintenance therapy. At this time, hydroxychloroquine 200 mg daily was reintroduced and added to his existing regimen of methotrexate and iguratimod, given his stable RA remission. Notably, throughout the entire CIDP disease course (from July 2024 to April 2026), the patient’s RA remained in sustained clinical remission, with consecutive monitoring showing ESR (ranging from 8 to 15 mm/h) and CRP (consistently <5 mg/L) within normal ranges and no recurrence of articular symptoms. This indicates that the neurological disease and articular disease evolved independently at the clinical level.

Two weeks post-discharge, the patient experienced significant worsening of ptosis and limb weakness, prompting re-admission on September 6, 2024. A repeat nerve conduction study, performed on September 7, 2024, now revealed new abnormalities. As shown in **Table 1**, the repeat NCS demonstrated abnormalities in two motor nerves (left ulnar and left median) meeting the 2021 EAN/PNS electrodiagnostic criteria for possible CIDP, characterized by mild conduction velocity slowing and reduced distal compound muscle action potential (CMAP) amplitudes, with all distal latencies and F-wave latencies remaining within normal limits. These findings, indicative of demyelination in at least two nerves, provided electrophysiological support for the diagnosis of A-CIDP. Given the progression of weakness beyond four weeks from symptom onset, the inadequate therapeutic response to initial treatment, and the evolving electrophysiological evidence of demyelination, the diagnosis was revised to A-CIDP.

**Table 1:** Serial nerve conduction study findings and fulfillment of the 2021 EAN/PNS electrodiagnostic criteria for CIDP.

Nerve	Parameter	2024-08-09	2024-09-07	Normal value	Meets EAN/PNS 2021 criterion
**Left Ulnar (Motor)**	**Distal latency (ms)**	**2.6**	**2.4**	**< 4.2**	**No**
	**Motor conduction velocity (m/s)**	**56.4**	52.5	**> 53**	Yes (slowing)
	**Distal CMAP amplitude (mV)**	**10.4**	6.4	**> 3.0**	Yes (reduction)
	**F-wave latency (ms)**	**Normal (value NR)**	**28.01**	**< 36**	**No**
**Left Median (Motor)**	**Distal latency (ms)**	**3.2**	**3.0**	**< 4.2**	**No**
**Motor conduction velocity (m/s)**	**63.2**	**54.5**	**> 50**	**No**	
	**Distal CMAP amplitude (mV)**	**8.4**	5.8	**> 5.0**	Yes (reduction)
	**F-wave latency (ms)**	**Normal (value NR)**	**25.96**	**< 33**	**No**
**Right ulnar (motor)**	**Distal latency (ms)**	**2.7**	**2.3**	**< 4.2**	**No**
	**Motor conduction velocity (m/s)**	**56.4**	**58.5**	**> 53**	**No**
	**Distal CMAP amplitude (mV)**	**9.1**	**8.0**	**> 3.0**	**No**
	**F-wave latency (ms)**	**Normal (value NR)**	**26.90**	**< 36**	**No**
**Left tibial (motor)**	**Distal latency (ms)**	**4.0**	**3.5**	**< 6.1**	**No**
	**Motor conduction velocity (m/s)**	**50.0**	**46.8**	**> 35**	**No**
	**Distal CMAP amplitude (mV)**	**8.9**	**8.8**	**> 3.0**	**No**
	**F-wave latency (ms)**	**Normal (value NR)**	**44.91**	**< 61**	**No**

CMAP, compound muscle action potential; NR, not reported; EAN/PNS, European Academy of Neurology/Peripheral Nerve Society.

Abnormal values supportive of demyelination are shown in bold.

According to the 2021 EAN/PNS guidelines, electrodiagnostic support for CIDP requires at least two nerves showing definitive demyelinating abnormalities (motor conduction velocity slowing, distal CMAP amplitude reduction, distal latency prolongation, or F-wave prolongation) in the absence of significant conduction block. In this patient, the left ulnar nerve demonstrated both conduction velocity slowing and CMAP amplitude reduction, while the left median nerve showed CMAP amplitude reduction. These findings in two nerves meet the criteria for electrophysiologically supported CIDP (possible CIDP category).

In light of this revised diagnosis, the patient received two doses of efgartigimod (1200 mg intravenously on September 9 and 13, 2024), resulting in only partial and transient improvement in facial muscle strength and limb power. Given the suboptimal clinical response and subsequent early relapse, the treatment strategy was reconsidered, and further doses were not administered. He was discharged on September 13, 2024, on prednisone 50 mg daily with a planned taper. However, in October 2024, he experienced a further relapse with worsening ptosis, prominent facial diplegia, masticatory weakness, and limb weakness, necessitating a third hospitalization. Intravenous methylprednisolone pulse therapy was again administered (500 mg/day for 2 days, tapered over 5 days) with modest benefit. Given the relapsing-remitting course and inadequate response to both IVIG and efgartigimod, MMF was introduced as a steroid-sparing immunosuppressant in April 2025 at a dose of 0.75 g twice daily, at which point prednisone had been tapered from 50 mg to 20 mg daily, but further reduction had repeatedly triggered worsening of weakness. Under this initial MMF dose (0.75 g twice daily), prednisone was gradually tapered from 20 mg to 10 mg daily over the following months, without symptom recurrence. In October 2025, MMF was up-titrated to 1 g twice daily to consolidate the therapeutic response and to allow for potential further tapering in the future, and prednisone was successfully maintained at 10 mg daily, thereafter, combined with methotrexate, iguratimod, and hydroxychloroquine. Under this intensified combined immunosuppressive regimen, the patient demonstrated sustained neurological improvement. Although not formally quantified using validated instruments, retrospective estimation based on physical examination records during hospitalizations and follow-up visits suggested that the patient’s Medical Research Council (MRC) sum score improved from approximately 48/70 at nadir (September 2024) to approximately 68/70 by April 2026, with the most pronounced improvement in proximal lower limb strength (from 4/5 to 5-/5) and facial muscle function. Correspondingly, the modified Rankin Scale (mRS) score improved from 4 (requiring assistance with walking) to 1 (no significant disability). By April 2026, only residual mild ptosis persisted, and no functional limitations were reported. **Figure 1** provides a chronological overview of the clinical course, key therapeutic interventions, and relevant laboratory trends from the time of CIDP onset through the final follow-up. As shown in the figure, the patient experienced three distinct relapses between August and October 2024, each occurring during corticosteroid tapering; it was only after the introduction of MMF in April 2025 that sustained, stable corticosteroid reduction without relapse was achieved.

**Figure 1:**
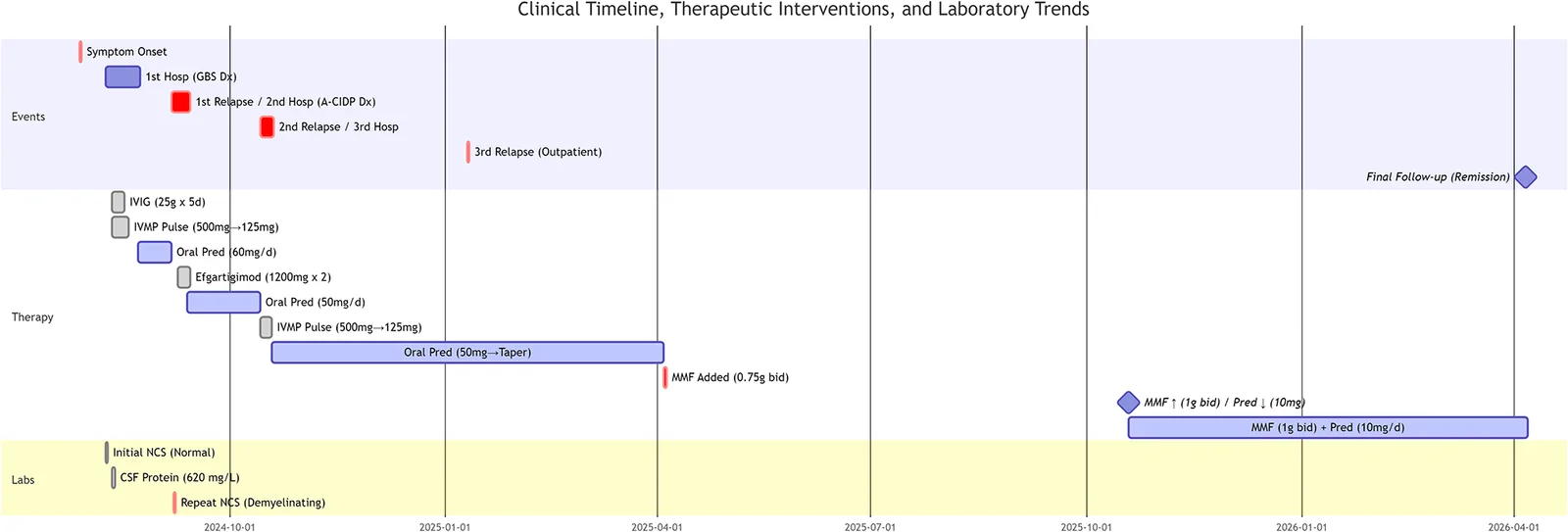
Clinical timeline, therapeutic interventions, and laboratory trends. This image illustrates the patient’s clinical course from symptom onset (July 29, 2024) to the final follow-up (April 6, 2026). The timeline integrates key clinical events (hospitalizations, relapses), therapeutic interventions (IVIG, corticosteroids, efgartigimod, MMF), and laboratory findings (NCS, CSF protein) in a chronological, color-coded manner. Red bars (crit) indicate critical events, including the three distinct relapses occurring during corticosteroid tapering between August and October 2024. Gray bars (done) denote completed short-term treatments. Blue bars (active) represent ongoing maintenance therapies. Diamond markers (milestone) signify important clinical milestones. Sustained clinical improvement and successful glucocorticoid reduction without relapse were achieved only after the introduction of MMF in April 2025. By the final follow-up, the patient remained on MMF 1 g twice daily and prednisone 10 mg daily, with only residual mild ptosis and no functional limitations. A-CIDP, acute-onset chronic inflammatory demyelinating polyradiculoneuropathy; CSF, cerebrospinal fluid; Dx, diagnosis; GBS, Guillain-Barré syndrome; HCQ, hydroxychloroquine; IGU, iguratimod; IVIG, intravenous immunoglobulin; IVMP, intravenous methylprednisolone; Labs, laboratory investigations; MMF, mycophenolate mofetil; MTX, methotrexate; NCS, nerve conduction study; Pred, prednisone; RA, rheumatoid arthritis; Therapy, therapeutic interventions.

## 3 Patient perspective

The patient described the acute, rapidly progressive weakness and facial droop as extremely distressing. Losing the ability to chew, speak clearly, and close his eyes within days caused intense psychological distress. He experienced deep frustration during the initial treatment period when he perceived minimal improvement despite intensive immunomodulatory therapy. The repeated hospitalizations and relapses were emotionally and physically draining. However, he emphasized that the gradual but sustained recovery following the introduction of MMF brought him significant relief and restored his hope for the future. He expressed sincere gratitude that he ultimately regained full independence and was able to return to work and daily life without major functional limitations, though he remains aware of the need for long-term treatment adherence.

## 4 Discussion

### 4.1 Coexistence of two immune-mediated diseases: RA and CIDP

This case illustrates three intersecting clinical challenges: the co-occurrence of two distinct immune-mediated diseases—RA and CIDP—in a single patient; the diagnostic evolution from an initial misdiagnosis of GBS to A-CIDP; and the therapeutic dilemma posed by a relapsing-remitting disease course that was refractory to both first-line immunomodulatory therapy and a novel targeted biologic, ultimately necessitating intensified conventional immunosuppression before clinical stabilization was achieved.

CIDP is a rare acquired immune-mediated neuropathy of the peripheral nervous system with an estimated prevalence of approximately 0.7 to 10.3 per 100,000 population and a reported incidence of less than 1 per 100,000 per year worldwide (5). The coexistence of CIDP with systemic rheumatic diseases, including RA and SLE, has been documented but remains exceedingly uncommon (4, 19). A 2017 case report described Lewis–Sumner syndrome, a multifocal variant of CIDP, developing almost concurrently with RA in a patient, raising the question of shared immunopathological mechanisms (3). CIDP has also been reported in association with SLE, Sjögren’s syndrome, systemic sclerosis, and mixed connective tissue disease (4, 19, 20). The pathophysiological basis for this association likely reflects the concept of autoimmune tautology, wherein individuals with one autoimmune disease harbor an increased susceptibility to developing a second immune-mediated condition owing to shared genetic predispositions—including certain human leukocyte antigen (HLA) haplotypes—and overlapping dysregulation of both T-cell and B-cell compartments (12, 13).

In the present case, the temporal sequence strongly implicates a shared autoimmune diathesis. The patient had seropositive, erosive RA that had been in sustained clinical remission for three years on a stable maintenance regimen of methotrexate and iguratimod. The development of CIDP in this context did not coincide with a flare of articular disease, indicating that the two autoimmune processes, while likely mechanistically related, were clinically independent. The observation that RA remission was preserved throughout the entire CIDP disease course further suggests that the underlying immunological tolerance was selectively breached at the level of the peripheral nerve, without generalized loss of immune homeostasis.

### 4.2 Diagnostic revision: from Guillain-Barré syndrome to A-CIDP

A critical aspect of this case is the diagnostic revision from GBS to A-CIDP. GBS is classically defined as a monophasic illness reaching its nadir within four weeks of symptom onset (18, 21), whereas CIDP is characterized by progressive or relapsing weakness exceeding eight weeks (7). The 2021 EAN/PNS guidelines provide updated diagnostic criteria for CIDP and its variants, emphasizing the integration of clinical course, electrophysiological evidence of demyelination, and supportive findings such as albuminocytologic dissociation in the CSF (7). In this patient, the initial nerve conduction study performed on day 12 of neurological symptoms was largely unremarkable—the only abnormality being an equivocal low-frequency decrement on right facial nerve repetitive stimulation. Coupled with the acute onset, preceding cosmetic injection, and marked CSF albuminocytologic dissociation (CSF protein 620 mg/L), the clinical picture was consistent with GBS (18). Treatment with IVIG was appropriately initiated as first-line therapy for suspected GBS (18). In view of the diagnostic uncertainty between GBS and A-CIDP, pulsed methylprednisolone was added to provide coverage for CIDP, for which corticosteroids are an established treatment (7).

However, the clinical trajectory diverged from that expected for GBS. The patient failed to demonstrate the typical plateau and gradual recovery phase (18); instead, weakness persisted and, after a brief partial improvement, worsened again approximately five weeks after symptom onset. A repeat nerve conduction study performed at re-admission revealed evolving electrophysiological abnormalities—mildly slowed left ulnar motor conduction velocity, reduced compound muscle action potential amplitudes in left median and ulnar nerves, and mildly reduced left ulnar sensory nerve action potential amplitude—consistent with an acquired demyelinating polyradiculoneuropathy (7). According to the 2021 EAN/PNS electrodiagnostic criteria, abnormalities in two motor nerves (left ulnar: conduction velocity slowing plus CMAP amplitude reduction; left median: CMAP amplitude reduction) meet the criteria for electrophysiologically supported ‘possible CIDP’—the lowest level of electrodiagnostic support. Although the electrophysiological threshold for ‘definite’ or ‘probable’ CIDP was not reached, the overall chain of evidence, integrating the clinical course exceeding eight weeks, the relapsing-remitting pattern, and CSF albuminocytologic dissociation, strongly supports the clinical diagnosis of A-CIDP. This electrophysiological limitation also reflects the dynamic evolutionary nature of nerve conduction studies in early A-CIDP and underscores the critical value of serial electrodiagnostic testing. The progression of weakness beyond four weeks, the relapsing-remitting pattern, and the emergence of definitive demyelinating features on electrodiagnostic testing collectively warranted the revision of the diagnosis to A-CIDP (7, 18).

It is important to distinguish A-CIDP from GBS with treatment-related fluctuations (TRF). While GBS is typically monophasic, reaching its nadir within four weeks (22–24)】, A-CIDP is characterized by a progressive or relapsing course exceeding eight weeks (22, 23, 25). In our patient, the clinical trajectory—with symptom onset on July 29, 2024, a partial response to initial therapy, and a significant relapse in October 2024—extended well beyond the eight-week threshold and followed a relapsing-remitting pattern. This protracted and fluctuating course, combined with the evolution of electrophysiological abnormalities, strongly supports the diagnosis of A-CIDP over GBS with TRF (22, 23).

This diagnostic evolution highlights an important clinical lesson: in patients presenting with an acute GBS-like illness that demonstrates inadequate therapeutic response or progression beyond four weeks, clinicians should maintain a high index of suspicion for A-CIDP and pursue serial electrodiagnostic studies (7, 26).

### 4.3 Challenges with first-line and targeted therapies

The treatment challenges in this case warrant detailed consideration. IVIG and corticosteroids are established first-line therapies for CIDP, with approximately 60% to 80% of patients demonstrating symptomatic improvement after receiving one of these modalities (9–11, 27). Plasma exchange is also a guideline-supported first-line option with comparable efficacy to IVIG. In our patient, this modality was not pursued because of poor peripheral venous access, the patient’s strong preference to avoid central venous catheterization, and the clinical team’s consideration that combined IVIG and corticosteroid therapy would provide broader immunomodulatory coverage during the period of diagnostic uncertainty between GBS and A-CIDP. However, a subset of patients—particularly those with acute-onset variants and certain antibody-defined subtypes—exhibit suboptimal responses or become dependent on high-dose corticosteroids, necessitating the introduction of steroid-sparing immunosuppressive agents (10, 11). In this patient, the initial combination of IVIG and high-dose methylprednisolone produced only marginal subjective improvement. Furthermore, multiple attempts at corticosteroid tapering were accompanied by relapses, a pattern consistent with the concept of glucocorticoid dependence frequently encountered in severe, treatment-resistant CIDP (10, 11).

Efgartigimod alfa, a first-in-class neonatal Fc receptor (FcRn) antagonist, represents a novel therapeutic strategy for CIDP (28, 29). By blocking FcRn-mediated IgG recycling, efgartigimod accelerates the degradation of circulating IgG, including pathogenic autoantibodies, thereby reducing IgG levels by approximately 50–60% (28, 29). The pivotal ADHERE trial established its efficacy in CIDP: among patients who responded to open-label efgartigimod in the run-in phase, those randomized to continued treatment had a 61% lower risk of clinical deterioration compared to those randomized to placebo (28). A pre-specified subpopulation analysis from ADHERE further demonstrated consistent efficacy and safety in Chinese participants (30).

However, in the present case, two doses of efgartigimod (1200 mg intravenously on September 9 and 13, 2024) produced only partial and transient improvement in facial strength and limb power, with a clinically significant relapse occurring within weeks. The decision to withhold further efgartigimod doses after the initial two infusions was pragmatic, based on the lack of a meaningful clinical response and the occurrence of early relapse, which suggested that continued treatment was unlikely to confer additional benefit. This approach reflects the clinical reality of managing severe, refractory CIDP, where timely recognition of treatment failure is critical to avoid delaying the initiation of potentially more effective alternative immunosuppressive strategies. Several factors may account for this inadequate response. First, CIDP is a heterogeneous disorder with diverse underlying immunopathogenetic mechanisms (6, 8). Although autoantibody-mediated pathways directed against paranodal proteins (e.g., neurofascin-155, contactin-1) and nodal proteins (e.g., neurofascin-140/186) are pathogenic in distinct CIDP subsets—collectively termed autoimmune nodopathies—a substantial proportion of patients with typical CIDP lack identifiable pathogenic autoantibodies, and T-cell-mediated mechanisms are believed to play a prominent role (8, 31). The efficacy of FcRn antagonism is predicated on reducing pathogenic IgG autoantibody levels; if the dominant effector mechanism is T-cell-driven or mediated by antibodies of the IgM class, the therapeutic impact of IgG depletion may be limited (6, 8). In this patient, the only autoantibody identified was GM4-IgG by immunoblot, an antibody of uncertain pathogenic significance that has been reported in GBS, Miller-Fisher syndrome, and multifocal motor neuropathy but is not a recognized biomarker of classic CIDP (8, 31, 32).

Second, the patient’s concurrent but clinically inactive RA may have contributed to a broader immunological milieu that was insufficiently targeted by isolated IgG depletion. RA is characterized by sustained activation of both T and B lymphocytes, with ongoing production of pro-inflammatory cytokines including tumor necrosis factor-alpha, interleukin-6, and interleukin-1β (1, 2). Even in the absence of overt articular inflammation, the immunological memory and chronic low-grade immune activation characteristic of treated autoimmune disease may render CIDP more treatment-resistant (1, 2, 12).

### 4.4 MMF as a core steroid-sparing strategy and clinical turning point

Following the failure of efgartigimod and the third CIDP relapse, MMF was introduced in April 2025 at 0.75 g twice daily and subsequently up-titrated to 1 g twice daily by October 2025. MMF is a potent inhibitor of inosine monophosphate dehydrogenase, the rate-limiting enzyme in *de novo* purine synthesis (33). Lymphocytes are uniquely dependent on this pathway, rendering MMF a relatively selective inhibitor of both T- and B-lymphocyte proliferation (33). In CIDP, MMF has been utilized as a steroid-sparing agent and has been associated with improvements in clinical outcome measures and reductions in IVIG utilization (11, 34). A 2025 retrospective case-control study demonstrated that the addition of MMF to IVIG with or without corticosteroids in patients with treatment-resistant CIDP was associated with significant improvements in the Medical Research Council Sum Score (median 58 to 68.5, p = 0.012) and the Inflammatory Rasch-built Overall Disability Scale score (median 47 to 78, p = 0.028), along with meaningful reductions in day-care unit utilization from 2.89 days/month to 1.65 days/month (p = 0.042), at a target MMF dose of 1–1.5 g twice daily over a 12-month treatment period (34).

Notably, the introduction of MMF marked a turning point in the patient’s clinical trajectory. It is important to recognize that MMF was added to an existing immunosuppressive background of methotrexate, iguratimod, and hydroxychloroquine, which had been maintained for RA. Therefore, the observed benefit reflects the contribution of MMF as an add-on steroid-sparing agent within a multi-target regimen, rather than a monotherapy effect. Prior to MMF initiation, each attempt to taper prednisone below 20 mg daily precipitated worsening of weakness, reflecting glucocorticoid dependence. MMF was introduced at this juncture and enabled further glucocorticoid tapering from 20 mg to 10 mg daily without relapse. By April 2026, on a combined regimen of MMF 1 g twice daily, prednisone 10 mg daily, methotrexate, iguratimod, and hydroxychloroquine, the patient was asymptomatic with only residual mild ptosis. This outcome underscores the therapeutic principle that, for CIDP patients with relapsing-remitting disease refractory to first-line agents and targeted biologics, intensification with conventional immunosuppressive agents that simultaneously target T- and B-cell pathways may represent an effective salvage strategy (11, 33, 34). Furthermore, the multi-target immunosuppressive regimen provided coverage for both CIDP and RA without necessitating additional therapeutic complexity—a practical advantage in managing patients with multiple autoimmune conditions (2, 11, 12).

### 4.5 Limitations and clinical implications

This report has several important limitations. First, as a single-case observation, causality cannot be definitively established, and the findings are not generalizable to broader populations. Second, detailed autoantibody profiling for CIDP-specific paranodal and nodal antibodies (e.g., anti-neurofascin-155, anti-contactin-1, anti-contactin-associated protein 1) was not performed, limiting the ability to classify the patient’s CIDP subtype and to predict treatment responsiveness with greater precision. Third, the temporal association between the facial cosmetic injections and CIDP onset cannot conclusively establish or exclude a causal relationship; the injections may have served as an immunological trigger or may have been entirely coincidental. Fourth, although we were able to derive retrospective estimates of the MRC sum score and mRS from the medical records, standardized neurological functional scales—including the MRC sum score, Inflammatory Neuropathy Cause and Treatment (INCAT) disability scale, and Inflammatory Rasch-built Overall Disability Scale (I-RODS)—were not prospectively or systematically applied at predefined time points. This limits the objective quantification of clinical improvement and precludes a detailed, validated longitudinal assessment of treatment response, as recommended by the 2021 EAN/PNS guidelines. Nevertheless, the sustained functional recovery, the ability to walk independently, and the successful glucocorticoid tapering without relapse provide clinically meaningful, albeit retrospective and semi-quantitative, evidence of benefit. Fifth, the follow-up period, although extending to April 2026 (21 months from disease onset and 12 months after MMF initiation), remains limited in the context of a chronic, potentially relapsing disease, and longer-term surveillance is required to ascertain the durability of remission. Sixth, the absence of specific serological biomarkers for monitoring CIDP disease activity limits the ability to objectively assess subclinical disease activity during treatment.

Despite these limitations, this case generates several clinically relevant hypotheses. It suggests that the presence of multiple autoimmune conditions may identify a subset of CIDP patients with more treatment-refractory disease, potentially warranting earlier and more aggressive immunosuppressive intervention. It further demonstrates that MMF, as part of a multi-agent immunosuppressive strategy, can serve as an effective steroid-sparing agent in CIDP, enabling successful glucocorticoid tapering even after an inadequate response to a short course of FcRn-targeted therapy. Prospective studies are warranted to validate these observations and to elucidate the optimal therapeutic sequencing in CIDP patients with comorbid autoimmune rheumatic diseases.

In summary, this single case report provides hypothesis-generating evidence that, for a patient with RA-associated, treatment-refractory A-CIDP, a multi-target conventional immunosuppressive regimen anchored by MMF was associated with sustained disease remission and successful glucocorticoid tapering. This observation highlights the need for further prospective studies to validate the efficacy and safety of this approach in larger cohorts. Furthermore, this case underscores the clinical importance of vigilant diagnostic reassessment in patients with pre-existing autoimmune disease who present with atypical or evolving neuropathies.

## Data Availability

The raw data supporting the conclusions of this article will be made available by the authors, without undue reservation.

## References

[1] Smolen JS Aletaha D Barton A Burmester GR Emery P Firestein GS . Rheumatoid arthritis. Nat Rev Dis Primers. (2018) 4:18001. doi: 10.1038/nrdp.2018.1 29417936

[2] McInnes IB Schett G . The pathogenesis of rheumatoid arthritis. N Engl J Med. (2011) 365:2205–19. doi: 10.1056/NEJMra1004965 22150039

[3] Kedra J Foltz V Viala K Tan S Fautrel B . Lewis-Sumner syndrome in a patient with rheumatoid arthritis: Link between rheumatoid arthritis and demyelinating polyradiculoneuropathies. Joint Bone Spine. (2017) 84:485–7. doi: 10.1016/j.jbspin.2017.02.013 28363822

[4] Julio PR Cortês MMM Costallat LTL Gaino JZ França MC Jr. Appenzeller S . Chronic inflammatory demyelinating polyradiculoneuropathy associated with systemic lupus erythematosus. Semin Arthritis Rheum. (2021) 51:158–65. doi: 10.1016/j.semarthrit.2020.09.018 33383292

[5] Broers MC Bunschoten C Nieboer D Lingsma HF Jacobs BC . Incidence and prevalence of chronic inflammatory demyelinating polyradiculoneuropathy: A systematic review and meta-analysis. Neuroepidemiology. (2019) 52:161–72. doi: 10.1159/000494291 30669140 PMC6518865

[6] Mathey EK Park SB Hughes RA Pollard JD Armati PJ Barnett MH . Chronic inflammatory demyelinating polyradiculoneuropathy: From pathology to phenotype. J Neurol Neurosurg Psychiatry. (2015) 86:973–85. doi: 10.1136/jnnp-2014-309697 25677463 PMC4552934

[7] Van den Bergh PYK van Doorn PA Hadden RDM Avau B Vankrunkelsven P Allen JA . European Academy of Neurology/Peripheral Nerve Society guideline on diagnosis and treatment of chronic inflammatory demyelinating polyradiculoneuropathy: Report of a joint Task Force-second revision. Eur J Neurol. (2021) 28:3556–83. doi: 10.1111/ene.14959 34327760

[8] Querol L Devaux J Rojas-Garcia R Illa I . Autoantibodies in chronic inflammatory neuropathies: Diagnostic and therapeutic implications. Nat Rev Neurol. (2017) 13:533–47. doi: 10.1038/nrneurol.2017.84 28708133

[9] Eftimov F Winer JB Vermeulen M de Haan R van Schaik IN . Intravenous immunoglobulin for chronic inflammatory demyelinating polyradiculoneuropathy. Cochrane Database Syst Rev. (2013) 2(2):Cd001797. doi: 10.1002/14651858.CD001797.pub3 24379104

[10] Kiers L Cruse B . Evaluation and treatment of refractory chronic inflammatory demyelinating polyradiculoneuropathy. BMJ Neurol Open. (2025) 7:e001318. doi: 10.1136/bmjno-2025-001318 41322367 PMC12658512

[11] Rajabally YA . Chronic inflammatory demyelinating polyradiculoneuropathy: Current therapeutic approaches and future outlooks. Immunotargets Ther. (2024) 13:99–110. doi: 10.2147/itt.S388151 38435981 PMC10906673

[12] Anaya JM . The autoimmune tautology. Arthritis Res Ther. (2010) 12:147. doi: 10.1186/ar3175 21092150 PMC3046506

[13] Richard-Miceli C Criswell LA . Emerging patterns of genetic overlap across autoimmune disorders. Genome Med. (2012) 4:6. doi: 10.1186/gm305 22284131 PMC3334554

[14] Cao X Guo J Yang Y Yu Z Pan H Zhou W . Clinical characteristics of Guillain-Barré syndrome in patients with primary Sjögren's syndrome. Sci Rep. (2024) 14:5783. doi: 10.1038/s41598-024-56365-y 38461210 PMC10924922

[15] Xiong A Cui H Deng R Wei X . Cyclophosphamide in the treatment of systemic lupus erythematosus-related Guillain-Barré syndrome: A systematic review of case reports. J Neuroimmune Pharmacol. (2023) 18:285–93. doi: 10.1007/s11481-023-10075-w 37391567

[16] Saied Z Zouari R Rachdi A Nabli F Ben Mohamed D Ben Sassi S . Cerebral large vessels vasculitis following Guillain-Barré syndrome as first clinical manifestations of primary Sjogren's syndrome: A case based - review. Heliyon. (2024) 10:e30004. doi: 10.1016/j.heliyon.2024.e30004 38698975 PMC11064453

[17] Ripellino P Schreiner B Latorre D . Expanding our understanding of Guillain-Barré syndrome: Recent advances and clinical implications. Eur J Immunol. (2024) 54:e2250336. doi: 10.1002/eji.202250336 39188201

[18] van Doorn PA Ruts L Jacobs BC . Clinical features, pathogenesis, and treatment of Guillain-Barré syndrome. Lancet Neurol. (2008) 7:939–50. doi: 10.1016/s1474-4422(08)70215-1 18848313

[19] Li X Wang Y . Systemic lupus erythematosus with acute inflammatory demyelinating polyneuropathy: A case report and review of the literature. J Clin Med Res. (2016) 8:555–9. doi: 10.14740/jocmr2550w 27298667 PMC4894028

[20] Birnbaum J Petri M Thompson R Izbudak I Kerr D . Distinct subtypes of myelitis in systemic lupus erythematosus. Arthritis Rheum. (2009) 60:3378–87. doi: 10.1002/art.24937 19877037

[21] Dimachkie MM Barohn RJ . Guillain-Barré syndrome and variants. Neurol Clin. (2013) 31:491–510. doi: 10.1016/j.ncl.2013.01.005 23642721 PMC3939842

[22] Inan B Bekircan-Kurt CE Demirci M Erdem-Ozdamar S Tan E . Differentiating recurrent Guillain-Barre syndrome and acute-onset chronic inflammatory polyneuropathy: Literature review. Acta Neurol Belg. (2024) 124:1467–75. doi: 10.1007/s13760-024-02557-2 38664341

[23] van den Berg B Walgaard C Drenthen J Fokke C Jacobs BC van Doorn PA . Guillain-Barré syndrome: Pathogenesis, diagnosis, treatment and prognosis. Nat Rev Neurol. (2014) 10:469–82. doi: 10.1038/nrneurol.2014.121 25023340

[24] Weiss N Marois C Le Guennec L Rohaut B Demeret S . Critical insights for intensivists on Guillain-Barré syndrome. Ann Intensive Care. (2025) 15:67. doi: 10.1186/s13613-025-01464-w 40394364 PMC12092332

[25] Rałowska-Gmoch W Koszewicz M Łabuz-Roszak B Budrewicz S Dziadkowiak E . Diagnostic criteria and therapeutic implications of rapid-onset demyelinating polyneuropathies. Exp Mol Pathol. (2024) 140:104942. doi: 10.1016/j.yexmp.2024.104942 39500252

[26] Rajabally YA Stettner M Kieseier BC Hartung HP Malik RA . CIDP and other inflammatory neuropathies in diabetes - diagnosis and management. Nat Rev Neurol. (2017) 13:599–611. doi: 10.1038/nrneurol.2017.123 28914883

[27] Hughes RA Mehndiratta MM Rajabally YA . Corticosteroids for chronic inflammatory demyelinating polyradiculoneuropathy. Cochrane Database Syst Rev. (2017) 11:Cd002062. doi: 10.1002/14651858.CD002062.pub4 29185258 PMC6747552

[28] Allen JA Lin J Basta I Dysgaard T Eggers C Guptill JT . Safety, tolerability, and efficacy of subcutaneous efgartigimod in patients with chronic inflammatory demyelinating polyradiculoneuropathy (ADHERE): A multicentre, randomised-withdrawal, double-blind, placebo-controlled, phase 2 trial. Lancet Neurol. (2024) 23:1013–24. doi: 10.1016/s1474-4422(24)00309-0 39304241

[29] Howard JF Jr. Bril V Vu T Karam C Peric S Margania T . Safety, efficacy, and tolerability of efgartigimod in patients with generalised myasthenia gravis (ADAPT): A multicentre, randomised, placebo-controlled, phase 3 trial. Lancet Neurol. (2021) 20:526–36. doi: 10.1016/s1474-4422(21)00159-9 34146511

[30] Lin J Ding M Wang Q Chang T Zhao Y Da Y . Efficacy, safety, and tolerability of subcutaneous efgartigimod PH20 in Chinese patients with chronic inflammatory demyelinating polyneuropathy: A prespecified subpopulation analysis of the multicentre, randomised-withdrawal, double-blind, placebo-controlled, phase II ADHERE trial. J Clin Neurol. (2026) 22:76–88. doi: 10.3988/jcn.2025.0247 41517815 PMC12802028

[31] Querol L Siles AM Alba-Rovira R Jáuregui A Devaux J Faivre-Sarrailh C . Antibodies against peripheral nerve antigens in chronic inflammatory demyelinating polyradiculoneuropathy. Sci Rep. (2017) 7:14411. doi: 10.1038/s41598-017-14853-4 29089585 PMC5663697

[32] Willison HJ Yuki N . Peripheral neuropathies and anti-glycolipid antibodies. Brain. (2002) 125:2591–625. doi: 10.1093/brain/awf272 12429589

[33] Allison AC Eugui EM . Mycophenolate mofetil and its mechanisms of action. Immunopharmacology. (2000) 47:85–118. doi: 10.1016/s0162-3109(00)00188-0 10878285

[34] Miah S Japzon N Elsaddig A Koay S Compton L Lunn MP . Mycophenolate facilitates improvement in outcome measures in treatment resistant chronic inflammatory demyelinating polyradiculoneuropathy. Muscle Nerve. (2025) 72:250–7. doi: 10.1002/mus.28436 40357692

